# “I finally found a place where I could have some safety”: A mixed-methods evaluation of non-clinical safe spaces for emotional distress and/or suicidal crisis

**DOI:** 10.1371/journal.pmen.0000572

**Published:** 2026-03-20

**Authors:** Scott J. Fitzpatrick, Grenville Rose, Heather Lamb, Cassandra Chakouch, Alyssa R. Morse, Amelia Gulliver, Helen T. Oni, Alison L. Calear, Michelle Banfield

**Affiliations:** 1 Centre for Mental Health Research, The Australian National University, Australian Capital Territory, Australia; 2 Black Dog Institute, University of New South Wales, Sydney, Australia; 3 Sonder, Adelaide, Australia; 4 College of Medicine and Public Health, Flinders University, Adelaide, Australia; 5 ALIVE National Centre for Mental Health Research Translation, Melbourne, Australia; Public Library of Science, UNITED STATES OF AMERICA

## Abstract

‘Safe Spaces’ are novel interventions that provide non–clinical, peer–led services for people experiencing emotional distress and/or suicidal crisis. To date, little empirical research has examined these service models, particularly from the perspective of those accessing and using them. Led by a team of lived experience researchers and using a convergent mixed-methods study design, this study examined whether safe spaces are a feasible and effective approach to supporting people experiencing varying degrees of emotional distress, including a suicidal crisis. Results showed that multiple features of safe space models, including the physical environment and approach to care taken by peer workers, had considerable therapeutic value, resulting in improved outcomes and reduced distress for guests. However, growing demand for services coupled with restricted resourcing and the implementation of protocols for managing guest entry and exit had implications for the perceived quality of care and how guests experienced the service. While safe spaces mark a transformative shift in the provision of care and support for people experiencing emotional distress and/or suicidal crisis, greater resourcing and attention to personal models of recovery are needed to maximise the benefits that can be gained from this model of care.

## Introduction

Over recent decades, a growing body of research has shown a poor fit between the support needs of those experiencing emotional distress, suicidal thoughts, and self-harm and the care provided by hospital emergency departments (ED). In addition to environmental and systemic issues associated with EDs such as waiting times, privacy, sensory stimuli, and superficial assessment procedures, inappropriate staff behaviour and ineffective treatment options have been identified as reasons for poor service-user experiences and outcomes [1–3]. People who visit the ED for mental health-related issues often experience longer wait times before being assessed, remain in the ED longer for care, and are more likely to leave without adequate aftercare [4,5]. Interactions with EDs have been shown to reinforce feelings of shame and distress, leading to further self-harm and discouraging future help-seeking [6].

In response to these concerns, Australian federal, state and territory governments, service providers, and suicide prevention and lived experience organisations identified the need for alternative, non–clinical, and peer–led service models [7–9]. The term ‘safe space’ (also referred to as safe haven) broadly refers to non–clinical, peer–led support services for people in suicidal distress [10]. The National Safe Spaces Network Scoping Study outlined five tiers of safe space models involving a range of supports across different settings – for example, those operated and co-located on health precincts, through to those that are community-based or volunteer-led [9]. Evidence from similar service models operating internationally and locally, in conjunction with the national scoping study that assessed overall feasibility, supported the initial roll out of services in partnership with state and territory governments and following local co-design activities with people with lived experience [9]. Since 2020, there has been a progressive roll-out of these services across Australia dependent on federal and state and territory government funding [10].

The emergence of safe spaces coincides with a wider recognition within the suicide prevention sector of the importance of integrating lived experience perspectives into service design, delivery, and evaluation [11]. Safe spaces operate as standalone services delivered by government or government-funded organisations with experience in the provision of health and social services. They provide face-to-face support and are led and delivered by peer workers with personal lived experience of suicide, either through their own personal experience of emotional distress and/or suicidal crisis, or through supporting a family member through this experience. They provide an alternative to emergency departments for people experiencing emotional distress and/or suicidal crisis through provision of a calm and ‘safe’ environment where people can receive peer-to-peer support, participate in activities to ease distress, and where necessary be connected to other services or supports that address their diverse needs [9]. The capacity of peer workers to establish rapport and meaningful connections through shared lived experience is a critical factor in effectively responding to the needs of individuals experiencing emotional distress or suicidal crisis, as is the creation of a home like, sensory-friendly environment [9]. The minimum training requirement for suicide prevention peer workers is a Certificate IV in Mental Health Peer Work, with additional training dependent on organisational policies and resourcing. The Certificate IV is a 12-month full-time course. Core areas of competency include applying lived experience in mental health peer work, working effectively in trauma informed care, and assessing and promoting social, emotional, and physical wellbeing.

To date, there has been little research conducted in Australia or internationally to examine the experiences of safe space service users. Service evaluations suggest that safe spaces improve the experience of care provision and social connections within the local community, and that they play an important role in helping service users to de-escalate an immediate crisis or to prevent a crisis from escalating [8,12]. Key features of service models valued by service users included respectful and compassionate care provision in a welcoming environment with no long waits and the opportunity to re-visit as needed [13]. Further benefits included the provision of preventative care and support to help service users maintain mental health and wellbeing and the economic benefits of reduced ED presentations [12,13]. ‘Guests’ is the preferred term to describe people accessing a safe space for support.

This study seeks to build upon these findings to examine whether safe spaces are a feasible and effective approach to supporting people experiencing varying degrees of emotional distress including a suicidal crisis through a specific focus on the experiences of those who access safe spaces for support. The current study aims to assess the feasibility and effectiveness of safe spaces by addressing the following research questions: i) How do guests experience the service? ii) What aspects of the service are most beneficial to guests? iii) To what extent are their needs met by the service? Through answering these questions, this study provides important insights for policy and practice into the design and implementation of safe space models, successes and challenges, and the processes needed to improve the experiences and outcomes of guests.

## Methods

### Setting and participants

The study was conducted across six safe space services in three Australian jurisdictions: Australian Capital Territory (ACT), New South Wales (NSW), and South Australia (SA). Study sites were selected based on well–established relationships between the researchers, mental health services, and suicide prevention networks and their interest in building the evidence base for safe spaces. Of the six services, two were entirely funded and delivered by government health services, while four were commissioned by government-funded suicide prevention collaborations and delivered by non-government organisations experienced in the provision of health and social services. The six services were positioned in a range of different settings including residential housing, shopping precincts, and community services buildings. Settings were structured by the different built environments in which they existed and ranged from open partitioned rooms through to spaces with a mix of communal and private spaces. Study participants were those aged 16 years and over who had accessed a safe space for support. Safe spaces have an open-door policy with all walk-ins accepted. One visit was sufficient for study participation.

### Study design

This study is part of the Co-Creating Safe Spaces project, a large multisite study of newly developed safe spaces in Australia. It involved a core team of academic researchers (including researchers with lived experience of suicide), health and community service managers, peer workers, and lived experience advocates. It was designed to embed co‐design into all aspects and stages of research from conception through to dissemination. For further details on research co-design see Fitzpatrick et al. [14,15].

We used a convergent mixed methods study design, collecting both quantitative and qualitative data from safe space guests. Data collection methods and key topics for interview and survey questions were developed in a series of co-design workshops involving the core team and safe space staff from the study sites. Merging was consistent with an exploratory unidirectional approach whereby findings from the interview data were used to guide analysis of the survey data and enhance interpretation of the merged data [16]. All data were collected between June 2023 and June 2024. For full study details see the published research protocol [17].

### Ethical considerations

The ethical aspects of this study were approved by the ACT Health Human Research Ethics Committee (Reference 2022.ETH.00043). To acknowledge the contributions of other lived experience experts, we offered participants the choice to decide if their interview data was presented under their own names, pseudonyms, or whether no attribution was made. Participants were informed of the potential risks of disclosing their identities. Pseudonyms are designated by an asterisk, while those who chose no attribution have been labelled as ‘Guest’. Informed consent was obtained from all participants. Approval for oral consent was granted for participants who took part in online interviews. Oral consent was recorded.

### Data collection

Our approach to data collection was guided by the need to balance the demands for data to evaluate guest outcomes and experiences with guests’ needs for care and support [14]. To provide choice and agency, we offered participants a range of voluntary data collection methods including short and long surveys and semi-structured interviews. To this end, there was some overlap between questions in the surveys thereby enabling comparison of data outputs. Participants were notified about the study by safe space staff and via site-specific recruitment material located within the safe spaces such as flyers and postcards. These contained QR Codes for participants to access the surveys or to nominate their interest in participating in an interview. Digital tablets with pre-loaded surveys were used at four study sites. Participants were informed that they could complete one or more of the surveys at any time after their service visit to ensure they were comfortable and that the research did not interfere with their care. Interviews were scheduled at a time and location convenient to participants.

#### Qualitative data collection.

We conducted a total of 11 semi-structured interviews with nine safe space guests (female = 8, male = 1) from four of the six safe spaces. Three participants completed two interviews in line with the original purpose of using journey mapping methodology for understanding how guests enter, experience, and exit safe spaces. This was discontinued in the later part of the study as timelines were no longer sufficient to allow for multiple interviews. Two participants consented to being interviewed together following a scheduling conflict. Interviews were conducted by HL (ACT), CC (NSW), and SF (NSW) using a semi-structured interview guide (S1 Appendix). Interviews were largely participant directed, starting with a broad question about the experiences that led them to visit the safe space, with some prompting questions used to elicit information on service use, experiences, and needs (S1 Appendix). On average, interviews lasted approximately 40 minutes. Sample size decisions were made over the course of the project by the primary investigator (MB) based on interpretative, situated, and pragmatic judgements about time, resources, and the purpose and goals of the research, and in keeping with the concept of thematic analysis as “a situated, reflexive, and theoretically embedded practice of knowledge generation or construction, rather than discovery” [18]. Interviews were conducted either in person or remotely depending on participant preferences and were recorded using a digital voice recorder or the video conferencing platform Zoom. Interviews were transcribed using the Zoom audio transcription tool or Microsoft Office 365 followed by transcription review by a member of the project team to check for accuracy and amend any errors.

#### Quantitative data collection.

We used three digital surveys to assess the effectiveness of safe spaces: the Entry/Exit survey, the COGwheel (Co-Designed Outcomes for Guests), and the Online survey (S1 Appendix). The Entry/Exit survey comprised four questions related to distress upon entering and exiting the service that participants were asked to complete after their visit (from 0 = no distress to 10 = very high distress), satisfaction with the service (from 1 = very dissatisfied, to 5 very satisfied), and where guests would have accessed support if the safe space had not been available. The COGwheel is a brief, user-friendly digital instrument co-designed specifically for this study. It assessed the level to which participants felt distressed, welcome, safe, comfortable, heard, connected, and empowered after using the safe space on a scale of 1 (not at all) to 5 (very much). For a detailed description of the COGwheel see Giugni et al. [19]. The Online survey collected information on accessibility, service use, experiences, and effectiveness, in addition to questions about individuals’ distress and suicidality. This survey comprised 34 closed-response and five open-ended questions, with three questions focusing specifically on outcomes from a carer perspective. In addition, four questions asked about age, gender, and other categories of social identity including race, sexuality, and neurodivergence. Closed-response questions included a mix of Likert scale and multiple response questions. The estimated completion time of surveys was between two and four minutes for the Entry/Exit survey and COGwheel and between 15 and 45 minutes for the Online survey based on responses to open-ended questions. All survey questions were optional, enabling respondents to skip any questions they did not want to answer. Surveys were anonymous and delivered using the online platform Qualtrics.

At the end of each survey, participants were asked if they consented to being contacted to take part in an interview and an embedded link took them to a separate webpage that collected their personal information. Those who consented were contacted by the research team via their preferred method of contact and invited to participate in an interview. A separate QR Code specifically for interviews was available for those who did not wish to participate in a survey.

#### Data analysis.

***Qualitative data analysis:*** Qualitative data were analysed using Braun and Clarke’s [20] 6-phase model of reflexive thematic analysis in line with a realist method. Reflexive thematic analysis is a creative yet systematic approach to interpreting and coding qualitative data, emphasising the active role of researchers in theme development [20]. Themes are conceptualised as actively created by the researcher rather than topic or domain summaries that passively ‘emerge’ from the data [21]. Researcher positionality and experience is particularly relevant in the case of lived experience researchers representing the voices of others with lived experience. Interpretation was guided by lived experience perspectives, including shared understandings of distress, marginalisation, discrimination, and a respect for different views. A realist method treats what people say as reflecting their experiences, meanings, and reality [22]. Nevertheless, we also endeavoured to consider the way meaning was created socially and, in particular, certain cultural understandings about suicide and suicidal persons including prevailing conceptualisations of distress, risk, and recovery.

Data familiarisation and initial coding phases commenced after data collection was completed and involved the first author reading the transcripts multiple times to generate ideas about the data in response to the research questions and producing an initial list of codes. Coding was then performed manually to identify codes that captured important features of the data relevant to the research questions before labelling and collating extracts of data using NVivo 12. Through a recursive process of analysis and mapping, a series of preliminary themes were generated by the first author as a means of conceptualising and organising data patterns and relationships. This took the form of a table with the name and a brief description of each code used to organise, refine, and develop the set of preliminary themes. Following this, further checking of themes against the coded data and entire dataset was undertaken to ensure all relevant data were captured and that they directly addressed the research questions [22]. This involved the first author reading all the collated interview data to consider whether the preliminary themes formed a coherent pattern, and the validity of these themes in relation to the meanings in the dataset as a whole [22].

Preliminary themes were reviewed and refined by the first author to determine the focus and scope of each theme and to decide upon an informative name for each. This involved returning to the collated interview data and preparing a written description of what each theme captured and how they contributed to the overall account of the data [22]. This resulted in the generation of five themes: 1) Somewhere to be (and to be myself), 2) Service gaps and support for mental health service users, 3) Shared ways of understanding distress and suicidality, 4) Balancing diverse needs and safety, and 5) Recovery and social participation. Quantitative analysis further explored the experiences of guests regarding service use, accessibility, needs, and effectiveness. These findings were used to enhance results from the qualitative data and to contextualise themes in relation to barriers and facilitators of good care.

***Quantitative data analysis:*** All analyses for the Entry/Exit, COGwheel, and Online surveys were conducted using SPSS 29. Where participants were asked to ‘tick all that apply’, responses were analysed using multiple response tables. Guest’s experience of accessing the service was assessed by asking participants to rate aspects of their experiences using the service on a scale of 1 (Strongly Disagree) to 5 (Strongly Agree), these results were reported descriptively. In both the Entry/Exit and Online surveys any change in levels of distress between arriving at the service and after using the service was assessed by asking how participants felt before and after attending the service on a scale from 0 (No distress) to 10 (Very high distress). Statistical significance of any change on that scale was assessed using the Wilcoxon t non-parametric analysis which, while conservative, is most appropriate for small samples where it is difficult to assess normality [23]. The COGwheel survey of participants’ perceptions of the service was reported descriptively.

## Results

In this section we report survey and interview participants’ quantitative and qualitative responses. In total, 19 participants completed the Online survey, 19 the Entry/Exit survey, and 91 the COGwheel. The Online survey was the only instrument that asked about different aspects of social identity. A total of 44% (n = 8) of the respondents reported identifying as diverse across a range of options provided, including gender or sexually diverse, and neurodivergent. Culturally and linguistically diverse representation was lower than in the general population of the service areas at 5.6% (n = 1). Just over one-fifth of Online survey participants (22%, n = 4) identified as a carer for someone with a disability or an ongoing physical or mental illness. Just over 20% of guests were visiting a safe space for the first time, but there was a wide distribution of the number of visits with 50% of guests having visited 5 or fewer times and the complement having visited more than 6 times (Table 1).

**Table 1:** How many times have you visited the safe space?.

	Frequency	Percent
This was my first visit	4	22.2%
1 to 2 times	3	16.7%
3 to 5 times	2	11.1%
6 to 10 times	1	5.6%
11 to 15 times	2	11.1%
More than 15 times	6	33.3%
Total	18	100.0%

### Somewhere to be (and to be myself)

A majority of online survey participants (83%, n = 15) reported experiencing a high level of distress prior to attending the safe space, with 78% (n = 14) of people saying that they were coping with suicidal or difficult thoughts or emotions. Relationship issues (33%, n = 6), social rejection (28%, n = 5), and grief and loss (22%, n = 4) were also listed by a large proportion of people accessing the service, with each person listing on average five reasons for visiting safe spaces. The most important needs identified by guests were access to a quiet space and a sensory-friendly environment, with each of these listed by 78% of participants as important.

In accordance with this, interview participants also described the value of having a place they could escape to that was sheltered from some of the more negative aspects of home or public spaces.

[I] remember feeling like I found, I finally found a place where I could have some safety; like a safe space… My home wasn’t safe… the outside world wasn’t safe for me. So, I remember this being my literal only safe space to be able to come to where I didn’t have any negative… experiences or emotions or like attitudes. I could just be me without any judgement. [Sarah]

Warm, non-judgemental support is an important feature of safe spaces. As well as a place to be safe, interview participants described the importance of feeling accepted for who they were, without having to conceal aspects of their identity or negative emotions.

I feel like I can be myself. I’m worthy to be myself here. I don’t have to dim my light. I don’t have to change… I’m full on. I’m chaotic. And here that’s OK to be that way, and people accept me and welcome me and encourage that in me. [Rayen]

For several participants, this extended to simply being in the space without the need to talk or interact with others.

That’s another thing about [safe] haven, you’re not feeling rushed like in a clinical appointment or even like on a phone line where… you feel like if you can’t speak, then they just want to hang up on you because you’re not speaking. Whereas at [safe] haven… every single time I go there, the first half hour I don’t speak at all, and that’s, everybody knows that. They’ll come and check on you… and sometimes I will sit there for four hours, I won’t speak, and then I’ll leave and that’s fine. [Zara*].

While some interview participants indicated a strong preference for being left alone, for others the need to talk and share what they were feeling with others was important. A key component of the co-designed models of care, therefore, was giving guests the time and space to deal with their distress in the ways they best saw fit, be that sitting in silence, playing games, doing artwork, or talking with peer workers.

### Service gaps and support for mental health service users

For those who had previously sought care in the emergency department, safe spaces offered a viable and effective alternative, providing an environment more conducive to sitting with and ameliorating distress and crisis.

I actually attended an emergency department nearly every single day for quite a few years. And ever since safe haven has opened up, I wasn’t attending emergency services… It was probably creating less trauma. I wasn’t having police come and tackle me. I wasn’t having, like, the repeated stigma of nurses and stuff like that making me feel like shit. [Chris*]

Further, several interview participants also reported that safe spaces filled an important service gap and that they would not have accessed services otherwise, mainly due to a lack of service options and previous negative experiences with the mental health system.

I don’t go anywhere else because the only place you can go is hospital. And the way that I’m treated in hospital, even when I go there for my physical condition, is absolutely disgusting. [Sarah]

For those participants using existing mental health services, safe spaces provided an important form of support between appointments.

From May till the end of June I had six weeks in an inpatient admission and leading up to that time I wasn’t coping with the spaces between the professional support, so I would use this space as that, you know, ‘keep me going’ space. [Letitia*]

The Online survey showed that the way participants found out about safe spaces was varied. There appeared to be some recognition of safe spaces within the broader health system with 39% of Online survey participants informed of the service through contact with the health system. This was supported by results from the Entry/Exit survey. When asked to select the two most likely support options if the safe space had not been open that day, the most common response was that they would have reached out to a telephone-crisis line such as Lifeline (38.9%, n = 7). A slightly smaller proportion of participants responded that they would not have reached out for support (33.3%, n = 6), would have reached out to someone they know (33.3%, n = 6), or that they didn’t know what they would have done (27.8%, n = 5) (Table 2).

**Table 2:** Entry/Exit survey responses to the question “If the safe space wasn’t open today, what supports or services would you most likely have tried instead?”.

	N	Percent of responses^a^
Phoneline or support service like Lifeline or Beyondblue	7	38.9%
Someone I know (e.g., friend, carer, family member, mentor)	6	33.3%
I would not have reached out for support	6	33.3%
I don’t know	5	27.8%
Local mental health acute care or crisis team	2	11.1%
000/ Emergency Services	2	11.1%
Hospital Emergency Department	2	11.1%
Online text or online forum	2	11.1%
Peer worker or support worker	1	5.6%
**Total**	**33**	**183.3%**

^a^Total greater than 100% as multiple responses were permitted.

Nearly half of Online survey participants thought safe spaces should be open weekday or weekend evenings (47%, n = 9) followed by weekday and weekend afternoons (42%, n = 8), and almost one-third of participants thought they should be open 24/7 (32%, n = 6). Those interview participants who reported feeling most dissatisfied with existing opening hours were those with school–aged children who were unable to attend in the afternoon or early evening due to caring responsibilities.

### Shared ways of understanding distress and suicidality

The emergence of safe spaces signals a growing acceptance and willingness on the part of governments to embrace new ways of working in the field of suicide prevention. Primary amongst these is a shift away from risk averse medical models toward approaches that are non-clinical, respectful, and that protect the dignity and rights of service users. For most interview participants, this approach was a recognised and valued aspect of safe spaces, especially when it came to the disclosure of suicidal thoughts.

That is so freeing… [just having] somebody not overreact so that you can actually say the truth. Being able to talk about things like suicidal ideation means I’m less likely to have to act on it because part of that ideation is coming from desperately wanting to change things or deal with feelings, and so being able to express those feelings gives me the ‘OK, I can express these feelings’… If you thought that, ‘oh if I say this, I’m going to be admitted or sedated’, then you can’t actually express that. [Zara*]

For Zara*, the privileging of risk management over the exploration of suicidal thoughts was unhelpful and inauthentic, inappropriately centering care on reducing organisational and personal liability rather than on providing support.

[It] feels like… I’ll get in trouble or somebody else will panic if I talk about it. And so it always just feels like nobody actually cares, they’re just, ‘just don’t do it on my watch’ kind of attitude. Like even like the helplines, it’s like, you know, ‘please just sound happy when you hang up on me’.

For those experiencing ongoing and persistent suicidal thoughts, this may lead to a reluctance to speak and the withholding of important information. Being free of harmful stereotypes and discriminatory practices, therefore, was key to the establishment of meaningful therapeutic relations.

I’ve always been told that, like, I’m too much. I’m too difficult. I’m too dramatic, too much of a burden that I can’t be helped. Like always just shoved from service to service like never really having people like pay attention or want to help or acknowledge anything that I was going through. Whereas with [safe space] I’ve never had anything near that. [Sarah]

In contrast, the practice of peer support in which peer workers sought to build genuine and equal relationships with guests based on principles of autonomy, choice, and self-determination marked a clear point of difference from conventional crisis services. Such an approach focuses on empowering guests to make decisions about their own care and safety, something that is often overlooked in clinical encounters.

Something that they do well is just to listen and accept. They don’t challenge. They offer suggestions that you might find helpful or not helpful. But there’s no expectation that you follow those suggestions. [Letitia*]

**Fig 1:**
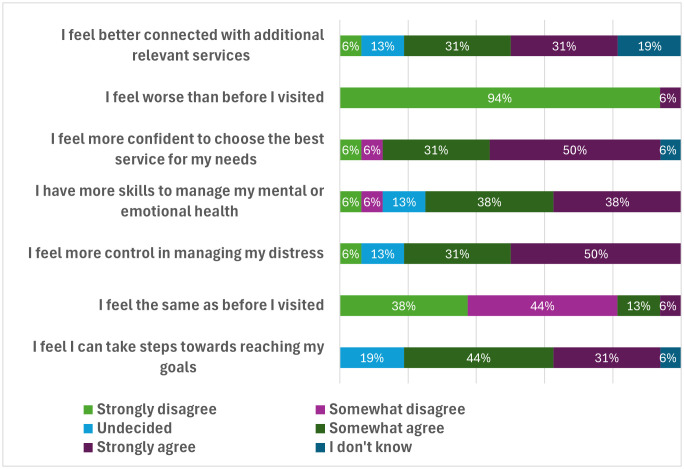
How participants felt during their visit to the safe space and outcomes from their visit, N = 19.

When asked how they felt the experience of accessing the service had benefited them, the vast majority on Online survey participants disagreed with the statement that they felt worse than before the visit (94%), or felt the same (82%), and the majority agreed that they could take steps towards reaching their goals (75%), felt more in control of their distress (81%), felt they had more skills to manage their mental and emotional health (76%), and felt better connected with additional services (62%) (Fig 1).

To this end, safe spaces were seen as providing a welcome alternative to the impersonal and procedural risk driven approaches of other service models, ensuring guests have appropriate choice and control over decisions about their care, and resulting in positive guest-centred outcomes.

### Balancing diverse needs and safety

**Fig 2:**
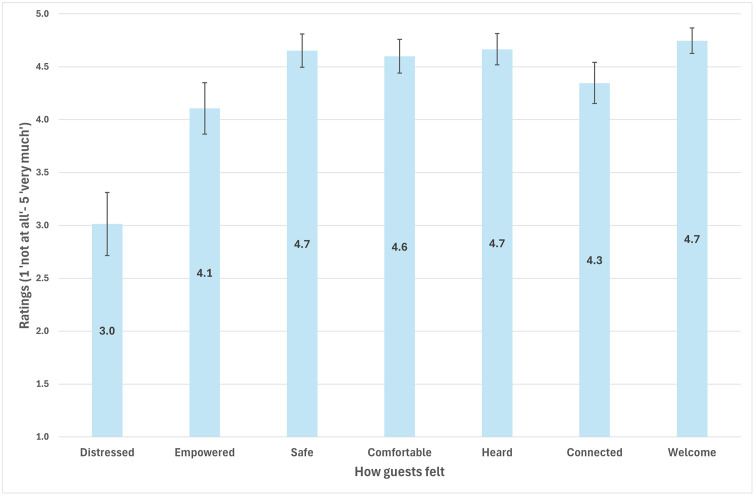
Mean scores on the COGwheel responses for guests’ experiences of safe spaces (95% confidence intervals), N = 91.

Balancing the varied needs of guests while maintaining a safe and supportive environment emerged as a central challenge for safe spaces. Key components of this balance were the characteristics and relational work of peer workers and service environments that were congruent with guests’ needs and preferences, which ranged from discussing their suicidality, distraction and sensory modulation, safety planning, social contact, and accessing referrals. Despite this diversity of needs, findings from the COGwheel showed that guests experienced safe spaces as welcoming, safe, and comfortable spaces where they felt heard (Fig 2).

In terms of improving feelings of safety for those guests experiencing suicidal distress, safe spaces were effective in helping guests manage and reduce their distress. On a scale from 0-10 where a higher number indicated higher distress, the Entry/Exit survey provided a retrospective measure of how distressed participants were when arriving at the service and after their visit. The median distress score on arrival was 8 and after attending was 3.5, which even with the small number of participants (N = 16) was statistically significant (Wilcoxon T_15_ = 3.53, *p* < 0.001).

Online survey participants were also asked how they felt about their experience while accessing the service in terms of specific relational elements of support and safety. They were asked 10 questions about positive aspects of their experience (e.g., I felt safe and comfortable with how staff responded to my emotions, I felt understood, Staff asked about my cultural beliefs of wellbeing and how I prefer to support myself) and 5 questions regarding negative aspects of their experience of the service (e.g., I felt judged or patronised by staff, I was made to feel like a burden to the service and outstaying my welcome, The visit didn’t seem helpful). On a scale of 1–5 the average positive score was 2.9 points higher than the average negative score and there was only one person whose average response about negative experiences was higher than their average regarding positive experiences.

The most significant contributor to guests’ sense of safety and support reported by interview participants was the interaction with individual peer workers. For most interview participants, these interactions were experienced as protective and stabilising during heightened periods of distress and vulnerability. Strong relational connections with peer workers were reported, with these relationships described using language that reflected the values and ethical principles of lived experience and peer work, including trust, respect, validation, genuineness, inclusivity, and non-judgement – qualities that linked directly to guests feeling safe to articulate their suicidal distress and ask for help, while having their own self-expertise acknowledged.

When you walk into Safe Haven… people treat you with dignity, with respect. They see a person before they see anything else. And they are so open and welcoming to who you are and what you want… They ask you, ‘how can I support you?… How can I walk alongside you? What do you need from us?’ [Rayen]

From this perspective safety was not seen simply as the reduction of harmful thoughts and behaviours, but as an active relational and collaborative process in which guests’ needs were recognised and prioritised. A safe space was one that enabled guests to articulate their needs when they felt least able to do so.

Unsurprisingly, some guests described bonding with some peer workers more than others, and there was evidence to indicate that close and enduring bonds between guests and peer workers were established across the multiple study sites with several participants reporting regular visits. Similarly, on a few occasions guests reported misunderstandings or miscommunication with peer workers or described how certain approaches adopted by them jarred with their needs and expectations.

I don’t know if they used, like, strategies, or asked me what strategies I have… I mean I just have a particular hatred for the whole tools or strategies thing as if there is absolutely no limit to what a human being can adapt to. I just feel like it just takes everything out of context and turns it into my, you know, unpreparedness to work hard enough to get better… I have to like prove that I’ve tried before I’ve, you know, dared to suck resources from somewhere. [Guest]

This resistance to the language of ‘strategies’ reflects a deeper discomfort with the implication that recovery from distress is simply a matter of effort or resourcefulness. Such framings can feel dismissive of the complexity of guests’ experiences and their reasons for seeking support. These tensions were echoed in broader reflections on the nature of safe spaces themselves. While intended as places of refuge and safety, interview participants described more nuanced and sometimes fraught experiences of safety and support that underscored the complexity of creating safety in shared environments. For instance, two participants noted the difficulty of avoiding other guests’ distress due to the relative lack of privacy in some safe spaces, which they found unsettling.

You can overhear most people, especially when they get stressed, they talk louder, and so that’s sometimes uncomfortable. [Zara*]

While safety was not an issue for one of the participants, for the other it was as it involved witnessing an intervention from emergency services with another guest which they found particularly distressing due to their own negative experiences of coercive interventions. The same participant described feeling uncomfortable with the gender balance during one visit, and particularly the limited number of female peer workers.

The access needs of guests and their ability to use the safe space in ways that were consistent with their needs and expectations also significantly impacted guests’ experiences of safety and the sense in which they felt welcome in the service. Participants described the challenges of navigating an increasingly overburdened safe space environment in which a higher volume of guest visits had begun to impact amenity at some safe spaces. To address the growing number of guests using safe spaces, time limits of two hours for guest visits had been introduced, and, in some cases, the closing of doors was required to manage the number of guests in the service at any one time. This had significant implications for some guests.

If it’s open but they’re full… that then causes a lot of feelings of, like feeling rejection, or feeling, you know, I’m not important enough to go in. But then also feeling guilt for feeling that, because obviously these other people deserve to be there too. And it creates a lot of feelings that are not that great that then add to whatever the reason I was going there in the first place. [Zara*]

Once in the safe space, Zara* described how she then spent considerable time worrying about someone knocking on the door and being asked to leave, and how that, at times, she felt obligated to tell staff she felt safe to leave when she really did not. These feelings of being rushed, pressured, and deprioritised ultimately undermined the sense of safety the service was intended to provide.

For Chris*, the introduction of these measures made access to the safe space more difficult than previously, leading him to wonder if new barriers and inequities were being created and to what extent he felt genuinely safe and supported.

Over time… safe haven has gone back on what they have said and began implementing restrictions like a two-hour limit when accessing the safe haven space. This was very difficult for me to adjust to when I was promised there would never be restrictions put in place at this service like I had experienced so many times before with mental health services that all have limitations. [Chris*]

The above quotes point to the critical challenge regarding the scope of safe spaces as they try to balance increasing pressure on staff and resources with guests’ and the broader communities’ need for safe crisis care and support, including recovery pathways and social participation.

### Recovery and social participation

Assumptions about personal responsibility and accountability for mental health and wellbeing extended to guests’ concerns about their experiences of distress, what it means to ‘recover’ from them, and the role of safe spaces in this process. From Morgan’s* perspective, there was a clear reluctance to revisit the safe space over personal concerns that they should have recovered by now.

I went back the one time, and I was really apprehensive to go back because I feel like even though I was not sure I bought into that like, linear healing line, I’m like, oh, it’s embarrassing and weird to go back. Like it’s supposed to be better. [Morgan*]

Yet it was also clear, from both the number of repeat guest visits and the descriptions provided by participants, that distress was often experienced as ongoing and enduring. Joanne, for example, described how visiting the safe space “resets your head” after even the deepest crisis, but how “at the same time, you feel like you’re walking out of here and then you’re going to go face this other world every time you walk back out.”

Amelia also attested to the struggles experienced by some participants who saw themselves as “broken’, “crazy”, or “destroyed” and the slow and difficult path to healing that many of them faced. For Amelia, the consistency of support and benefits of seeing regular faces was critical in helping her to try and rebuild her life. Attending further education, trying to establish a new career, and getting off the disability pension were all goals she attributed to her visits to the safe space. But she also understood that these required her to work on things herself and that she couldn’t lean too hard on safe spaces. Perhaps Sarah summed it up best when she said how it was nice to have someone in your corner when you most need it, reminding you “that [you] do have goals” and that there “are reasons to live”.

To what extent safe spaces were recovery-oriented in terms of additional aspects of personal recovery such as sense of belonging and social participation was not clear. This was reflected in the somewhat lower mean scores for feeling connected and empowered on the COGwheel (Fig 2) as well as interview responses. For example, one participant described how the set-up of their safe space provided little opportunity for guests to connect with other guests, while another claimed that interactions between guests were not supported at one site. However, for some participants the opportunity to build social connections within the local community was important in the larger sense of what it means to belong, feel safe, and be part of the community, elements key to personal recovery.

Even if it’s [safe space] not clinical it’s still one–on–one. I feel like… you just need more than two people to cue belonging in a bigger sense. I can feel safe with one person, but it doesn’t necessarily translate into me feeling safer, like in the world, or safer in the place I’m in. [Guest]

Views such as this challenge what should and should not fit within the scope of safe spaces, an area in which participants expressed some uncertainty in terms of differing service models and unmet community need.

## Discussion

This study has drawn on the perspectives of safe space guests to examine the feasibility and effectiveness of safe space models, to what extent they met guests’ needs, and those aspects of the service model that were most beneficial. The findings showed that most participants who used safe spaces reported high levels of distress upon seeking support that reduced as a result of their visit. Guests’ needs varied, but the majority described the need for a quiet space, a sensory-friendly environment, connection, and ongoing support. Safe spaces provided a viable and effective alternative to hospital emergency departments but also helped to fill important service gaps for those unwilling to seek formal support due to previous negative experiences of clinical services, or for those seeking a safe and supportive space in tandem with specialist and community mental health services.

Our findings provide support for early, emerging research on the effectiveness of peer–led, non–clinical services for those experiencing emotional distress and/or suicidal crisis. The provision of timely, non–judgemental, and compassionate care in a welcoming, safe, and non–clinical environment has been identified as a key ingredient of these services’ success [13]. Participants’ descriptions of safe spaces as a place to be and to be themselves demonstrates both the importance of the therapeutic environment in helping guests to articulate their distress, as well as its capacity to enable certain kinds of action and interaction that are supportive of guests’ needs. For those who have been harmed by clinical services or who suffer trauma, feelings of safety are a prerequisite for being able to participate, engage, and heal [24]. Life in the community is a challenge for many people living with serious and persistent mental illness and/or ongoing suicidal thoughts. Limited health and social service availability means it is increasingly likely that people will experience distress in environments that are not conducive to accommodating their needs [25]. Spaces that offer refuge, respite, and the opportunity to be with people who have encountered similar experiences without an underlying agenda of treatment have the potential to play an increasingly important and cost-effective role in assisting people in crisis [26–28]. This signals a need to shift away from risk management approaches to safety in crisis care toward those that take seriously its spatial, temporal, and relational dimensions [29].

Our findings are also consistent with research on the barriers confronting people wanting to use peer–led, non–clinical services, with restricted opening hours and capacity impacting service accessibility [13]. The implementation of protocols for managing increasing guest numbers had an adverse effect on some participants, presenting clear challenges to safety and the ability of peer workers to meet guests’ needs. These had the potential to impact guest outcomes and create new barriers and inequities. Extending opening hours was a key recommendation across previous studies [13]. Our findings indicate this was also the preference of some participants. At the time of writing, safe spaces operated between 4 and 5 days per week with hours extending from early afternoon to between 7:30pm and 10pm. Given that safe spaces are promoted as an alternative to the emergency department, additional funding to extend opening hours should be a key priority to expand access to these services. This is especially important in the context of our finding that one-third of participants stated they would not have reached out for support if the safe space was not open. Safe spaces, if adequately resourced, may be able to minimise some of the difficulties experienced by guests regarding continuing levels of access and support.

Our study provides new evidence on the characteristics and functions of peer–led, non–clinical service models. In addition to the need for a quiet space and a sensory-friendly environment, participants described the importance of guests and peer workers sharing similar ways of understanding distress and suicide. Being able to discuss suicide openly without fear of censure or unwanted coercive or clinical intervention was especially valued by participants. This finding highlights the importance of services being lived experience–designed and –led to ensure the core principles and knowledge of people with lived experience of suicide are embedded in practice. It also raises questions about the normalising of suicide. Research on the association between media reporting and suicide has long cautioned of the dangers of normalising suicide through open and honest conversation [30], while efforts to reduce stigma are sometimes seen as a double-edged sword, unintentionally normalising suicide and discouraging help seeking [31]. However, research on and by those with a lived experience of suicide has shown that stigmatisation and discrimination work to isolate and silence suicidal people, invalidating their experiences and preventing them from reaching out for support [32,33]. Rather than working from a preventionist and curative stance, recent critical scholarship advocates for a harm reduction approach anchored in the values of informed consent, self-determination, dignity of risk, and the right to refuse treatment [32]. In this view – one shared by participants in our study – normalising experiences of distress and the facilitating of open and curious conversations about suicide are key elements of safe space models, enabling those experiencing suicidality to talk about and work through complex issues, experiences, and emotions in a safe and supportive manner to receive the support they need.

As an alternative to the emergency department, safe spaces sit primarily within a crisis services model. However, findings from this study suggest there is a need from some segments of the community for social support, especially from those reporting complex and longstanding mental health issues where there are a shortage of social programs or services available. The National Safe Spaces Network Scoping Study recognised the role that peer workers play in personal recovery and maintenance of overall wellbeing [9]. The building of supportive social relationships is crucial to enhancing quality of life for those experiencing persistent struggles, social exclusion, loneliness, and discrimination [34]. Such perspectives point to the value of rethinking relational models of care within safe spaces to consider more collectivised models such as inclusive or recovery-oriented communities that foster interpersonal relationships beyond those of the guest–peer worker [35]. Extending the scope of practice to include the provision of regular group-based community activities might therefore provide important opportunities for connecting people, improving social networks, and fostering personal recovery [34].

## Strengths and limitations

This study has some limitations. The difficulty of sensitively collecting data from people in distress resulted in relatively small sample sizes that were likely not representative of all populations accessing safe spaces. Researchers and peer workers were mindful of the intrusive nature of certain lines of questioning around suicidality and their potential to cause distress. Differences in data collection procedures across study sites may also have rendered the study vulnerable to potential recruitment biases. Despite some overlap between questions, different survey instruments asked for slightly different information from guests. As these were not applied evenly across study sites, this may have yielded slight differences in results. However, the integration of quantitative and qualitative data from multiple sources and sites enabled robust comparison and triangulation of findings, generating important insights into guests’ service use, experiences, and needs. A further strength of the study was that it involved a core team of lived experience representatives, health and community service managers, and academic researchers, the majority of whom identified as having a lived experience of suicide. This meant that outcome measures and methods were all co-designed by stakeholders to assess acceptability and appropriateness prior to implementation of the research. Survey instruments were also reviewed by stakeholders to establish content and face validity.

## Conclusion

Safe spaces signal a transformative shift in the provision of care and support for people experiencing emotional distress and/or suicidal crisis. Faced with problems of access to timely and appropriate services, safe spaces have been designed and implemented to meet the specific needs of their intended users. This study showed that multiple features of the safe space model, including the physical environment and approach to care taken by peer workers, had considerable therapeutic value, resulting in improved outcomes and reduced distress for guests. Safe spaces provided a clear alternative to hospital emergency departments for some, yet they were also of particular benefit to people who did not otherwise seek help due to previous negative service experiences or unmet needs—a key suicide prevention measure. Growing demand coupled with restricted resourcing and the implementation of protocols for managing guest entry and exit had implications for the perceived quality of care and how guests experienced the service. While safe spaces provided a genuine alternative support pathway for people experiencing emotional distress and/or suicidal crisis, greater attention and focus on personal models of recovery are needed to maximise the benefits that can be gained from this model of care.

## Supporting information

S1 Appendix Survey instruments and interview guides. (PDF)
