# Supplementary material for: “I finally found a place where I could have some safety”: A mixed-methods evaluation of non-clinical safe spaces for emotional distress and/or suicidal crisis
Source: PLOS Ment Health. 2026 Mar 20;3(3):e0000572. doi: 10.1371/journal.pmen.0000572 (PMC13004489; doi:10.1371/journal.pmen.0000572)
Supplement: S1 Appendix — (PDF) [file pmen.0000572.s001.pdf]

ENTRY/EXIT SURVEY

Please indicate the level of distress you were feeling when you arrived at the Safe Haven.

Move the slider from 0 = no distress to 10 = very high distress.

- ○ 0
- ○ 1
- ○ 2
- ○ 3
- ○ 4
- ○ 5
- ○ 6
- ○ 7
- ○ 8
- ○ 9
- ○ 10

Please indicate the level of distress you were feeling immediately after visiting the Safe Haven.

Move the slider from 0 = no distress to 10 = very high distress.

- ○ 0
- ○ 1
- ○ 2
- ○ 3
- ○ 4
- ○ 5
- ○ 6
- ○ 7
- ○ 8

◯ 9

◯ 10

How would you describe your overall satisfaction with your experience at the Safe Haven today?

◯ Very dissatisfied

◯ Dissatisfied

◯ Neutral

◯ Satisfied

◯ Very satisfied

If the Safe Haven wasn't open today, what supports or services would you most likely have tried instead? Please choose the two most likely options.

◯ I would not have reached out for support

◯ Phoneline or support service like Lifeline or Beyondblue

◯ Online text or online forum

◯ Someone I know (e.g. friend, carer, family member, mentor)

◯ Peer worker or support worker

◯ GP

◯ Local mental health acute care or crisis team

◯ 000 / Emergency Services

◯ Hospital emergency department

◯ I don't know

<u>COGwheel[1]</u>

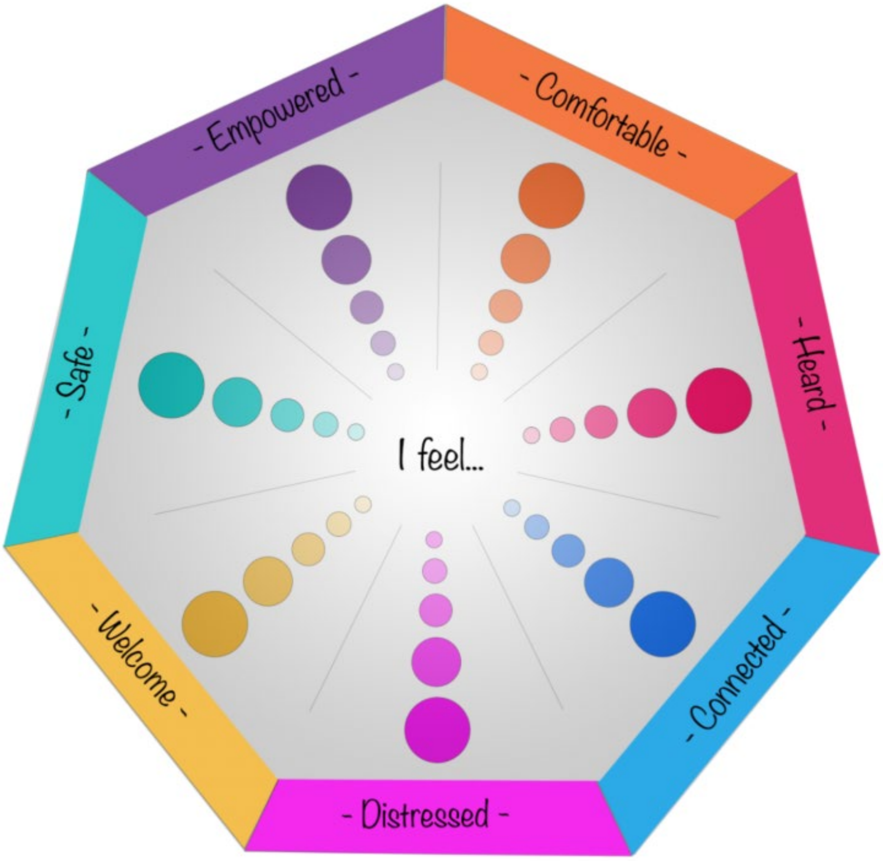

[1] Giugni M, Morse AR, Fitzpatrick SJ, Lamb H, Gulliver A, Calear AL, et al. Reinventing the evaluation wheel: COGwheel's co-designed digital innovation using the qualtrics heat map. MethodsX. 2025;14: 103147.

ONLINE SURVEY

Demographic information

The following questions help us understand who has taken part in our research:

1. What is your age?

2. What is your gender?

3. What is your postcode?

4. In recognising that individuals with overlapping and intersecting identities find it harder to access appropriate support, our research seeks to understand the experience of these individuals to help identify service gaps.

   If you are comfortable doing so, please select any of the following options which you feel apply to you:

- ○ BIPOC (Black, Indigenous, People of Colour)
- ○ CALD (Culturally and/or Linguistically Diverse)
- ○ Gender diverse
- ○ Sexually diverse
- ○ Neurodivergent
- ○ Autistic / on the Autism spectrum
- ○ Disabled / living with disability
- ○ Living with a chronic condition / chronic pain
- ○ Experiences mental distress / illness / ill-health
- ○ Carer for someone who lives with disability, is frail with age, or has an ongoing mental or other illness
- ○ Uses a language other than English at home
- ○ Experiences barriers related to alcohol and other drug use
- ○ Experiences barriers related to homelessness
- ○ Another group we have missed: (please specify)

Accessibility and use of the Safe Space

This survey is about the use of services known as a Safe Haven / Safe Space. When thinking about your answers, please think in terms of your most recent visit to the Safe Haven / Safe Space.

All questions are optional, just answer what you are comfortable with.

5. Please indicate the reason for your most recent visit to the Safe Haven / Safe Space. Choose all that apply.

○ Support or information for myself

○ Support or information as part of my unpaid family / friend caring role for another

○ Support or information as part of my paid / staff caring role for another

○ Visiting to see and learn about the service

○ Other ____

6. Which specific issues were you seeking support or information around? (Choose all that apply)

○ Distress / difficult or intense emotions

○ Thoughts of hurting myself or others

○ Suicidal thoughts

○ Mental health

○ Autistic burnout

○ Carer stress / fatigue

○ Social rejection / isolation

○ Relationships (friends / family / others)

○ Grief / loss

○ Domestic or family violence

○ Culture / Identity

○ Difficulty accessing appropriate support

○ Alcohol / other drugs

◯ Housing / Homelessness

◯ Education / Work / Unemployment

◯ Criminal justice

◯ Financial stress

◯ Physical health concerns

◯ Food or body image

◯ Sexuality or Gender

◯ Other(s): ____

7. How many times have you visited the Safe Haven / Safe Space?

◯ This was my first visit

◯ 1 to 2 times

◯ 3 to 5 times

◯ 6 to 10 times

◯ 11 to 15 times

◯ More than 15 times

8. How did you find out about the Safe Haven / Safe Space? Choose all that apply.

◯ I don’t know or can’t remember

◯ Someone just brought me there

◯ I’ve been there before

◯ Family/friend/carer told me about it

◯ Peer or support worker told me about it

◯ Caseworker / staff / doctor / clinician told me about it

◯ I saw a brochure or poster

◯ Online website or social media

◯ Hospital / Emergency Department

◯ Crisis team / police / ambulance

○ Other ____

9. When I engage with a service, it is important that I have (choose all that apply):

○ Quiet spaces

○ Sensory-friendly environment

○ Fragrance-free environment

○ Ability to bring a support person

○ Ability to bring a service animal

○ Physical Access (wheelchair, walker) for entry and bathrooms.

○ Large print, screen reader or Braille materials

○ Text captions on visual material

○ Attention to food allergies

○ Access to an interpreter

○ Other ____

○ None of these

○ Specify (for any of above)

____

10. To what extent were these needs met when you attended the Safe Haven?

	Definitely not	Probably not	Undecided	Probably yes	Definitely yes	Not applicable
Quiet spaces	○	○	○	○	○	○
Sensory-friendly environment	○	○	○	○	○	○
Fragrance-free environment	○	○	○	○	○	○

Ability to bring a support person	○	○	○	○	○	○
Ability to bring a service animal	○	○	○	○	○	○
Physical Access (wheelchair, walker) for entry and bathrooms.	○	○	○	○	○	○
Large print, screen reader or Braille materials	○	○	○	○	○	○
Text captions on visual material	○	○	○	○	○	○
Attention to food allergies	○	○	○	○	○	○
Access to an interpreter	○	○	○	○	○	○
Other	○	○	○	○	○	○
None of these	○	○	○	○	○	○

11. Please indicate who has been involved in you getting to the Safe Haven. Choose all that apply

	On my first visit, I went:	(If applicable) On other visits I have been:
By myself	○	○
With a friend or relative	○	○
With a peer worker or support worker	○	○
With a case worker or clinical staff member	○	○

Other	○	○

12. Please indicate how you have made your way to the Safe Haven / Safe Space. Choose all that apply.

- ○ Walking or bike
- ○ Public transport
- ○ Drove myself in car
- ○ Someone else drove me
- ○ Other ____

13. When do you think it is most important for the Safe Haven / Safe Space to be open? Choose all that apply.

- ○ Weekday Mornings
- ○ Weekday Afternoons
- ○ Weekday Evenings
- ○ Weekday Overnight
- ○ Weekend Mornings
- ○ Weekend Afternoons
- ○ Weekend Evenings
- ○ Weekends Overnight
- ○ All the time, 24/7
- ○ The most critical time is:

____

Diversion from Emergency Department

14. If the Safe Space / Safe Haven wasn't open today, what supports or services would you most likely have tried instead? Please choose the 2 most likely options.

◯ Peer worker or support worker

◯ An organised online peer support group or forum

◯ Formal phone/chat/text support service (e.g. Lifeline)

◯ Someone I know (e.g. friend, carer, family member, mentor)

◯ Local mental health acute care or crisis team

◯ 000 / Emergency Services

◯ Hospital Emergency Department

◯ GP (General Practitioner)

◯ Informal / social / anonymous online forum

◯ I would not have reached out for support

◯ I don't know

◯ Other (please specify)

15. Did you attend the Emergency Department (ED) shortly before your most recent visit to the Safe Haven / Safe Space?

◯ Yes

◯ No

◯ Prefer not to say

Skip To 19 If - No

Skip To 19 If - Prefer not to say

16. What prompted you to visit the Safe Haven / Safe Space following your attendance at the Emergency Department (ED)?

◯ ED staff suggested it

◯ Family / friend suggested it

○ I thought of it myself

○ Other ____

17. Did you attend the Emergency Department (ED) shortly after your most recent visit to the Safe Haven / Safe Space?

○ Yes

○ No

○ Prefer not to say

Skip To 19 If - No

Skip To 19 If - Prefer not to say

18. How did you come to attend the ED after visiting the Safe Haven / Safe Space? Choose all that apply.

○ Safe Haven / Safe Space staff suggested it

○ Family / friend suggested it

○ I decided myself

○ Other ____

<u>Experience and effectiveness of the Safe Space</u>

19. How were you feeling when you arrived at the Safe Haven / Safe Space?
Move the slider from 0 = no distress to 10 = very high distress.

○ 0

○ 1

○ 2

○ 3

○ 4

○ 5

○ 6

○ 7

- 8
- 9
- 10

20. After attending the Safe Haven / Safe Space, how were you feeling?
    Move the slider from 0 = no distress to 10 = very high distress

- 0
- 1
- 2
- 3
- 4
- 5
- 6
- 7
- 8
- 9
- 10

21. Please indicate how much you agree with these statements regarding how you felt during your visit to the Safe Haven / Safe Space.

	Disagree	Somewhat disagree	Undecided	Somewhat agree	Agree
I worried I would be shunned or avoided after disclosing certain information about myself	○	○	○	○	○
I felt judged or patronised by staff	○	○	○	○	○
I was made to feel like a burden to the service and	○	○	○	○	○

outstaying my welcome					
I felt staff were avoiding/trying to avoid dealing with me	○	○	○	○	○
I got the impression staff did not feel comfortable talking to me	○	○	○	○	○
My decisions were respected	○	○	○	○	○
I felt safe and comfortable with how staff responded to my emotions	○	○	○	○	○
Staff asked about my cultural beliefs of wellbeing, and how I prefer to support myself	○	○	○	○	○
Staff explained things in a way I could understand	○	○	○	○	○
I felt understood	○	○	○	○	○
Staff were knowledgeable about the issues I presented with	○	○	○	○	○
I felt accepted	○	○	○	○	○
Staff recognised me as the expert in my own life	○	○	○	○	○
Staff allowed and encouraged expression of any feelings (even "negative"	○	○	○	○	○

emotions) without judging me					
Staff made me feel welcome by being warm and friendly, and using a welcoming tone of voice	○	○	○	○	○
The visit didn't seem helpful	○	○	○	○	○

22. As a result of my visit to the Safe Haven / Safe Space:

	Disagree	Somewhat disagree	Undecided	Somewhat agree	Agree
I feel I can take steps towards reaching my goals	○	○	○	○	○
I feel the same as before I visited	○	○	○	○	○
I feel more control in managing my distress	○	○	○	○	○
I have more skills to manage my mental or emotional health	○	○	○	○	○
I feel more confident to choose the best service for my needs	○	○	○	○	○
I feel worse than before I visited	○	○	○	○	○

I feel better connected with additional relevant services	○	○	○	○	○

23. Please add any further comments, feedback or suggestions for improvements regarding your visit to the Safe Haven / Safe Space.

___

Exploring concept of safety from guest perspective

24. What makes a place or service feel safe for you?

___

25. Were there things you considered before deciding if it was safe to come to the Safe Haven / Safe Space?

___

26. What has been a "deal-breaker" or stopped you from returning to services?

___

Carer Perspective

People in a care relationship with someone in emotional distress or suicidal crisis experience their own journey and this is not always recognised. This set of questions invites you as (carer-family-supporter) to give your perspective on your visit to the Safe Haven in regards to your own needs.

27. Please indicate which of the following items relates to your visit/s to the Safe Haven (choose all that apply):

	On my first visit to the Safe Haven	On subsequent visits to the Safe Haven
I visited together with the person I care for	○	○

I visited without the person I care for	○	○
The purpose of my visit was mainly to accompany and support the person I care for	○	○
The purpose of my visit was mainly for my own needs	○	○
The purpose of my visit related to both myself and the person I care for	○	○

28. Whenever visiting the Safe Haven together with the person I care for, the Safe Haven staff:

	Disagree	Somewhat disagree	Undecided	Somewhat agree	Agree	Not relevant to me
Asked the person I care for what had brought us here today	○	○	○	○	○	○
Asked for my perspective on what had brought us here today	○	○	○	○	○	○
Supported the person I came with	○	○	○	○	○	○
Supported my own concerns or needs	○	○	○	○	○	○
Asked about my needs directly in front of the	○	○	○	○	○	○

person I was with						
Provided a discrete opportunity to talk with me separately	○	○	○	○	○	○
Made assumptions about my role in the care relationships	○	○	○	○	○	○
Asked about my ability, capacity and willingness to provide support	○	○	○	○	○	○

29. During my visit to the Safe Haven, staff were able to discuss or support me with:

	Disagree	Somewhat disagree	Undecided	Somewhat agree	Agree	Not relevant to me
Managing my own distress and strong emotions e.g. fear, anxiety, depression, anger, guilt	○	○	○	○	○	○
Managing loss and grief	○	○	○	○	○	○
My past exposure to the suicide attempts of others	○	○	○	○	○	○
My own history of suicidal	○	○	○	○	○	○

ideation or attempts						
Asked about my needs directly in front of the person I was with	○	○	○	○	○	○
My own feeling of safety	○	○	○	○	○	○
Balancing my own needs for support (disclosure to others) with my loved one’s privacy	○	○	○	○	○	○
How to keep the person I care for safe at home during a crisis	○	○	○	○	○	○
Decisions around how much supervision to provide at times of crisis	○	○	○	○	○	○
Information and advice on providing emotional support	○	○	○	○	○	○
Decisions on when and who to contact in a crisis	○	○	○	○	○	○
The dilemma of whether or not to initiate contact with services against a loved one’s wishes	○	○	○	○	○	○

The impact of a carer role on my life e.g. work, finance, lifestyle, relationships	◯	◯	◯	◯	◯	◯
Decisions around withdrawing support or ending the care relationship	◯	◯	◯	◯	◯	◯
Living with the consequences of my decisions	◯	◯	◯	◯	◯	◯
Self-care	◯	◯	◯	◯	◯	◯
Feeling encouraged about the future	◯	◯	◯	◯	◯	◯
Next steps for myself	◯	◯	◯	◯	◯	◯
Next steps for the person I care for	◯	◯	◯	◯	◯	◯

Suicide general questions

We recognise that the next set of questions may be particularly sensitive as they ask specifically about your history of suicidal thoughts or attempts. All questions are optional. Please be aware of how you are feeling and practice self-care strategies or contact a support person should you feel any discomfort. You can skip any question or SKIP the whole section by answering yes below.

Would you like to SKIP this section of questions about suicide?

◯ Yes

◯ No

Skip To 40 If - Yes

30. Have you had a close friend, colleague or family member take their own life?

- Yes
- No
- Prefer not to say

31. Do you personally support someone who experiences suicidal thoughts or has made a previous attempt on their life?

- Yes
- Unsure
- No
- Prefer not to say

Suicide questions adapted from CDC Youth Risk Behaviour Survey (YRBS) questions

32. During the past 12 months, did you ever consider suicide?

- Yes
- No

33. During the past 12 months, did you make a suicide plan?

- Yes
- No

34. During the past 12 months, how many times did you attempt suicide?

- 0 times
- 1 time
- 2 or 3 times
- 4 or 5 times
- 6 or more times

SIDAS - Suicidal ideation attributes scale

35. In the past month, how often have you had thoughts about suicide?
(0 = Never, 10 = Always)

- ◯ 0
- ◯ 1
- ◯ 2
- ◯ 3
- ◯ 4
- ◯ 5
- ◯ 6
- ◯ 7
- ◯ 8
- ◯ 9
- ◯ 10

Skip To 40 If - 0

36. In the past month, how much control have you had over these thoughts?
(0 = No control, 10 = Full control)

- ◯ 0
- ◯ 1
- ◯ 2
- ◯ 3
- ◯ 4
- ◯ 5
- ◯ 6
- ◯ 7
- ◯ 8
- ◯ 9
- ◯ 10

37. In the past month, how close have you come to making a suicide attempt?
(0 = Not close at all, 10 = Made an attempt)

- ○ 0
- ○ 1
- ○ 2
- ○ 3
- ○ 4
- ○ 5
- ○ 6
- ○ 7
- ○ 8
- ○ 9
- ○ 10

38. In the past month, to what extent have you felt tormented by thoughts about suicide?
(0 = Not at all, 10 = Extremely)

- ○ 0
- ○ 1
- ○ 2
- ○ 3
- ○ 4
- ○ 5
- ○ 6
- ○ 7
- ○ 8
- ○ 9
- ○ 10

39. In the past month, how much have thoughts about suicide interfered with your ability to carry out daily activities, such as work, household tasks or social activities? (0 = Not at all, 10 = Extremely)

- ○ 0
- ○ 1
- ○ 2
- ○ 3
- ○ 4
- ○ 5
- ○ 6
- ○ 7
- ○ 8
- ○ 9
- ○ 10

Distress - DQ5 Distress Questionnaire

40. In the last 30 days:

	Never	Rarely	Sometimes	Often	Always
My worries overwhelmed me	○	○	○	○	○
I felt hopeless	○	○	○	○	○
I found social situations upsetting	○	○	○	○	○
I had trouble staying focused on tasks	○	○	○	○	○
Anxiety or fear interfered with my ability to do	○	○	○	○	○

the things I need to do at work or at home	

Quality of life - ReQOL

41. For each of the following statements, please choose the option that best describes your thoughts, feelings and activities over the last week.

	None of the time	Only occasionally	Sometimes	Often	Most or all of the time
I found it difficult to get started with everyday tasks	○	○	○	○	○
I felt able to trust others	○	○	○	○	○
I felt unable to cope	○	○	○	○	○
I could do the things I wanted to do	○	○	○	○	○
I felt happy	○	○	○	○	○
I thought my life was not worth living	○	○	○	○	○
I enjoyed what I did	○	○	○	○	○
I felt hopeful about my future	○	○	○	○	○
I felt lonely	○	○	○	○	○
I felt confident in myself	○	○	○	○	○

42. Please describe your physical health (problems with pain, mobility, difficulties caring for yourself or feeling physically unwell) over the last week.

- ○ No problems
- ○ Slight problems
- ○ Moderate problems
- ○ Severe problems
- ○ Very severe problems

43. Thank you, you have now reached the end of the survey. Before you proceed to submit your responses, is there anything else you wish to add?

____

GUEST INTERVIEW GUIDE

Interview 1

1) Tell me about your experiences over the last week. What were the events that led you to come to the safe space?

Probe for:	Factors contributing to distress
	Why they sought support
Prompt:	How did you feel about accessing the safe space?
Probe for:	Personal barriers/enablers
	Social, cultural barriers/enablers
Prompt:	What were your needs and expectations of the safe space?
Probe for:	Care or support needs
	Safety
Prompt:	Tell me about your experience from when you arrived at the safe space till when you left?
Probe for:	Different phases of the journey through the service
Prompt:	What experiences stand out to you as helpful or unhelpful?
Probe for:	Access, communication, attitudes, model of care, staff
Prompt:	How do you feel about the care and support you were offered?
Probe for:	Did it meet needs and expectations? Why, why not?
Prompt:	How do you feel about the safe space staff member who supported you?

Probe for: Experience with peer worker, experience with clinician

Prompt: What changes to the service would make a difference for you?

Probe for: Communication, attitudes, behaviours, people

Prompt: Do you do anything new or different now after visiting the safe space?

Probe for: Changes in behaviour

Interview 2

1. Tell me about how you have been since we last talked?

Prompt: Have you attended the safe space on any other occasion?

Prompt: [How] did the safe space help you plan the next steps of your recovery?

What has it been like following those steps/taking the next steps for your recovery?

Probe for: Have you visited any other services since we talked? Have you used any other self-care strategies or social supports?

Prompt: [How] could the safe space have better helped you plan the next steps of your recovery?

Prompt: Follow up on any major issues or key areas of discussion from Interview 1

Prompt: What would you like your future recovery to look like?

Probe for: What supports/services would best help you achieve this?
